# A Self-Administered Multicomponent Web-Based Mental Health Intervention for the Mexican Population During the COVID-19 Pandemic: Protocol for a Randomized Controlled Trial

**DOI:** 10.2196/23117

**Published:** 2020-11-16

**Authors:** Alejandro Dominguez-Rodriguez, Anabel De La Rosa-Gómez, M Jesús Hernández Jiménez, Paulina Arenas-Landgrave, Sofía Cristina Martínez-Luna, Joabian Alvarez Silva, José Ernesto García Hernández, Carlos Arzola-Sánchez, Victoria Acosta Guzmán

**Affiliations:** 1 Valencian International University Valencia Spain; 2 Coordinación de Educación a Distancia Facultad de Estudios Superiores Iztacala Universidad Nacional Autónoma de México Mexico City Mexico; 3 Facultad de Psicología Universidad Nacional Autónoma de México Mexico City Mexico; 4 ITLAB Mexico Juarez Mexico; 5 Plan Estratégico de Juárez A C Juarez Mexico; 6 Institute of Social Sciences Autonomous University of Ciudad Juárez Juarez Mexico

**Keywords:** e-health, positive psychology, cognitive behavioral therapy, behavioral activation therapy, COVID-19, internet, intervention, telepsychology, Mexican sample

## Abstract

**Background:**

The COVID-19 pandemic has become a public health emergency of international concern; it has not only threatened people's physical health but has also affected their mental health and psychological well-being. It is necessary to develop and offer strategies to reduce the psychological impact of the outbreak and promote adaptive coping.

**Objective:**

This study protocol aims to describe a self-administered web-based intervention (Mental Health COVID-19) based on the principles of positive psychology supported by elements of cognitive behavioral therapy and behavioral activation therapy to reduce the symptoms of anxiety and depression and increase positive emotions and sleep quality during and after the COVID-19 outbreak through a telepsychology system.

**Methods:**

A randomized controlled clinical superiority trial with two independent groups will be performed, with intrasubject measures at four evaluation periods: pretest, posttest, 3-month follow-up, and 6-month follow-up. Participants will be randomly assigned to one of two groups: self-administered intervention with assistance via chat or self-administered intervention without assistance via chat. The total required sample size will be 166 participants (83 per group).

**Results:**

The clinical trial is ongoing. This protocol was approved by the Research Ethics Board of the Free School of Psychology-University of Behavioral Sciences (Escuela libre de Psicología-Universidad de Ciencias del Comportamiento). The aim is to publish the preliminary results in December 2020. A conservative approach will be adopted, and the size effect will be estimated using the Cohen *d* index with a significance level (α) of .05 (95% reliability) and a conventional 80% power statistic.

**Conclusions:**

The central mechanism of action will be to investigate the effectiveness of an intervention based on positive psychology through a web platform that can be delivered through computers and tablets, with content that has been rigorously contextualized to the Mexican culture to provide functional strategies to help the target users cope with the COVID-19 pandemic.

**Trial Registration:**

ClinicalTrials.gov NCT04468893; https://clinicaltrials.gov/ct2/show/NCT04468893

**International Registered Report Identifier (IRRID):**

DERR1-10.2196/23117

## Introduction

### Background

The outbreak of COVID-19 has been declared a Public Health Emergency of International Concern (PHEIC) [1]. It has also affected people’s mental health and has had consequences for their psychological well-being. In a study conducted in China to determine the impact of the initial phase of the COVID-19 outbreak on people’s mental health, more than half of the respondents evaluated the negative psychological impact of the outbreak as moderate or severe. Moreover, the participants reported depressive symptoms (16.5%), anxiety (28.8%), and moderate to severe stress levels (8.1%) [2]. Subsequently, increases in negative emotions (eg, anxiety, depression, and irritability) and sensitivity to social risks have been observed, as well as a decrease in positive emotions and life satisfaction after the official declaration of the epidemic of COVID-19 in China [3]. During this pandemic, public health measures have been implemented to mitigate the spread of the virus, such as physical distancing and confinement worldwide. However, although these measures can be critical to mitigate the spread of the disease in the general population, the separation from loved ones, the perception of loss, and the uncertainty of the evolution of the disease could cause adverse psychological effects both in the short and long term [4]. Due to the outbreak control measures, it has been observed that confinement, loss of normal routine, and reduction of social and physical contact with others frequently resulted in feelings of boredom, frustration, and isolation, which were perceived as distressing to the participants [5].

In Mexico, at the time of the writing of this manuscript, as of July 18, 2020, 324,041 cumulative cases of COVID-19 have been reported and 37,574 deaths have been confirmed [6], and it is estimated that the pandemic may continue to have devastating effects considering the structural conditions of poverty and lack of access to physical and psychological health assistance. Although published research articles about the impact of COVID-19 in Mexico are still scarce, it was identified that just one week after the national health emergency was declared in Mexico, 50.3% of a total sample of 1105 participants rated the psychological distress of the outbreak as moderate to severe, followed by 15.7% participants who reported moderate to severe depressive symptoms and 22.6% who reported moderate to severe anxiety symptoms [7]. Another study with a sample of 3932 participants identified that 14.8% of the participants reported moderate and 7.8% reported severe intrusive thoughts, 15.9% reported moderate and 6.4% reported severe avoidance symptoms, 9.8% reported moderate and 2.4% reported severe symptoms of hyperarousal, and 27.7% reported clinically significant symptoms of posttraumatic stress [8].

The pandemic presents a double challenge because not only is it necessary to design and develop interventions to meet the demand for mental health services, but these interventions must also be adapted to the requirements of a population that is currently in confinement and cannot attend in-person sessions to receive psychological support. Thus, the development of remote psychological care services that provide service to the general population is of extreme relevance. Therefore, there is an emerging need to develop cost-effective mental health prevention interventions, not only to cover the demand for care existing after the COVID-19 pandemic but also to reduce risk factors that increase the possibility of either developing mental health problems or exacerbating the symptoms of pre-existing mental disorders [9,10]. The purpose is to strengthen these factors with individual tools such as positive thinking, interpersonal effectiveness skills, and problem-solving [9], among other mechanisms that enable the general population to positively adapt to adversity. Positive psychology is one of the approaches that focuses on promoting such tools.

### Psychological Intervention Based on Positive Psychology

Positive psychology is a movement within psychology that strives to better understand meaning in life, character strengths, and how these can be developed [11]. It is defined as the scientific study of positive experiences, positive individual traits, and institutions that facilitate the development of these experiences and traits, as well as programs that improve the quality of life of individuals while preventing or reducing the incidence of psychopathology [12-14]. Positive psychology seeks to complement traditional psychology; it does not deny suffering and negative aspects in people and seeks to correct the imbalance that affects the homeostasis of daily life [15]. Individuals can intentionally strengthen their ability to experience and maximize positive emotions, which has been shown to improve their physical, emotional, and social health [16]. People are happier and have fewer depressive symptoms after receiving positive psychology [17]. Seligman et al [18] considered it necessary to distinguish at least three access routes to happiness: positive emotions and pleasure (pleasant life); commitment (committed life); and meaning (life with meaning). Furthermore, positive and negative affect usually exist in the same continuum [19]. Positive psychology is oriented toward the prevention and treatment of emotional problems such as anxiety, depression, and stress, among others [20-24]. The objective of the professionals who conduct positive psychology interventions with adults is to increase the emotional well-being of said adults [25].

### Efficacy of Interventions Based on Positive Psychology

The topics treated in interventions based on positive psychology, such as strengths, positive emotions, and emotional regulation, produce positive effects on happiness levels, therefore reducing worry, increasing the construction of personal resources, and improving general well-being [19,25,26]. Positive psychology interventions drive happiness through the activation of positive emotions [27], increase aspects of positive body image, and have a significant impact on health and well-being [28]. In interventions based on gratitude for aspects related to well-being and mental health, increments of subjective happiness and life satisfaction as well as reduction of negative affect and depression symptoms were observed [29]. Moreover, research into new approaches using positive psychology interventions is increasing, such as a randomized controlled trial with three groups [30]. Sin and Lyubomirsky [31] carried out a meta-analysis of 51 interventions involving 4266 participants, and the results revealed that positive psychology interventions did significantly improve well-being (mean *r*=0.29) and decrease depressive symptoms (mean *r*=0.31). The efficacy and effectiveness of positive interventions that aimed at cultivating pleasure, commitment, and meaning have also been demonstrated [32]. Although the efficacy of positive psychology has been studied during the last 40 years, it is necessary to be more exhaustive and include more studies in meta-analyses and better effect sizes. This will allow significant analyses to be performed to determine the efficacy of the various interventions based on positive psychology, particularly whether individual interventions are more effective than group interventions and whether longer interventions are more effective than shorter ones [33].

It is also interesting to note that cognitive behavioral therapy (CBT) and positive psychology are compatible, and sometimes one can nurture the other. Both approaches involve analyzing thoughts and behaviors while taking emotions into consideration, while always pursuing the psychological well-being of people [34]. Thus, it has been proven that the application of behavioral activation therapy can be an effective approach to reduce anxiety and depression because it can help people become reinvolved in their lives [35]. In addition, in some studies, it has been pointed out that behavioral activation therapy can be useful for the prevention and treatment of emotional disorders; it can modify dysfunctional patterns by increasing the involvement of the person in what is valuable for them and thus reinforce their efforts. It should be noted that there this research still involves heterogeneity and limitations, and there is insufficient evidence for its use as a sole therapeutic approach [35].

### Efficacy of Web-Based Positive Psychology Interventions to Enhance Mental Health in Adults

Web-based interventions through digital platforms offer the possibility of two-way communication and therapeutic approaches [36] and are recommended by official psychological colleges, such as the Official College of Psychology of Madrid. In their Guide to Telepsychological Intervention [37], they state that web-based interventions provide advantages such as accessibility to people who otherwise would not request psychological assistance; in addition to these factors and benefits, they provide quick and easy recording of information to justify the integration of information and communications technology into the therapeutic process. Similar recommendations can be found in the guides of the Colombian College of Psychologists [38] and the College of Professionals in Psychology of Costa Rica [39]. Web-based therapy is advantageous at times when it is difficult or complicated to attend a therapy center, such during as the COVID-19 outbreak; web-based approaches can help avoid the spread of the disease caused by the SARS-CoV-2 virus [40]. The internet can be useful to carry out self-administered psychological interventions [41] and enhance accessibility to therapy for all those who need it [42]. It is important to note that in different reviews, self-administered treatments via the internet and computer-based treatments have been found to be effective [43-45]. Studies have affirmed that the substantial effect of the intervention in positive psychology occurred when it is applied on the internet [46]. In addition, web-based positive psychology therapy with exercises designed to promote positive emotions, behavior, thoughts, strengths, and virtues was found to be effective in reducing symptoms related to depression or other emotional problems [47]. Furthermore, studies have evaluated web-based treatment programs for sleep disorders in adults [48], reporting a significant improvement in participants who received the intervention based on positive psychology [49,50]. Web-based positive psychology therapy has shown significant improvements in both well-being and depressive symptoms [51,52]; significant improvements were obtained in the life satisfaction and general well-being of the participants.

Technology plays a fundamental role in the transmission of positive attitudes. However, there is insufficient knowledge about the factors that influence acceptance and compliance with web-based interventions [53]. It is too early to draw conclusions regarding the psychological consequences of the COVID-19 outbreak in the population. Professionals are conducting research to determine the influencing factors, and as mentioned above, it is estimated that interventions in positive psychology can provide benefits and improve the well-being of the population during the COVID-19 outbreak.

The aim of this study is to describe a randomized controlled trial to evaluate the efficacy of a web-based self-administered positive psychology intervention program based on a telepsychology system (Mental Health COVID-19) for the reduction of anxious and depressive symptoms and the increase of positive emotions and sleep quality during and after the COVID-19 outbreak.

### Hypotheses

#### Primary Hypothesis

The self-administered web-based intervention with psychological assistance via chat will show greater statistical gains in the reduction of anxiety and depression symptoms and greater improvement in positive psychological functioning than an intervention program without support.

#### Secondary Hypotheses

Higher rates of acceptance and satisfaction will be reported by the participants in the web-based intervention program with psychological support via chat compared to the intervention without assistance; coping strategies and acceptance and satisfaction will be found to function as moderating variables of clinical change; and the changes will be maintained for three and six months after the end of the intervention programs with and without psychological support via chat.

## Methods

### Study Design

A randomized controlled study will be carried out according to the guidelines set forth in the Consolidated Standards of Reporting Trials (CONSORT) statement [54] and CONSORT eHealth checklist [55].

**Figure 1:**
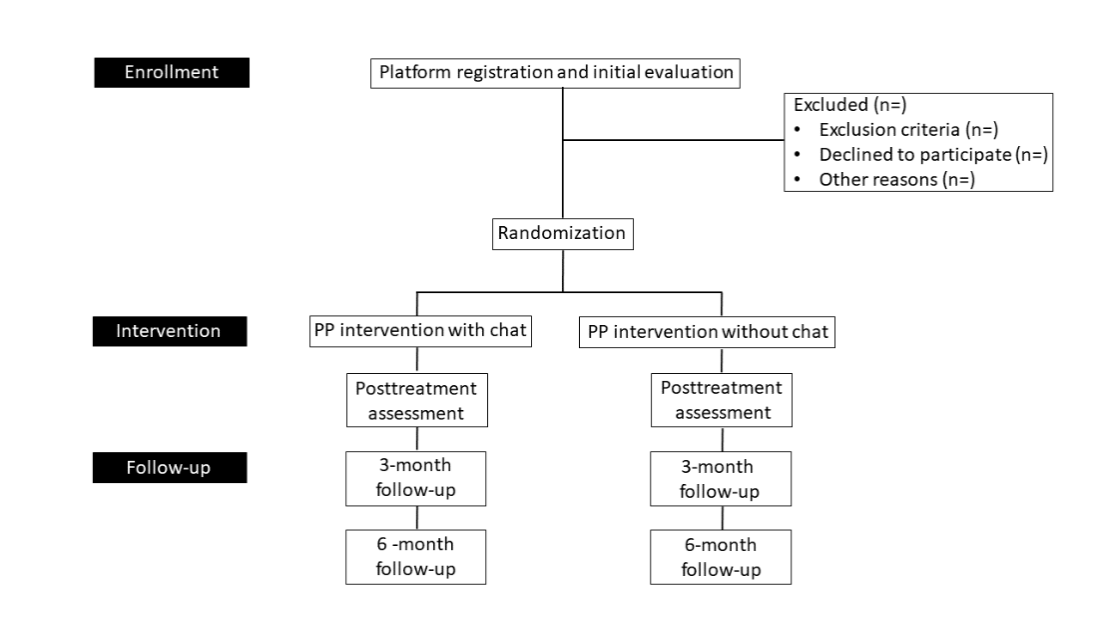
Flowchart of the study design for the Mental Health COVID-19 platform. PP: positive psychology.

A randomized controlled clinical superiority trial with two independent groups will be used, with intrasubject measures at four evaluation periods: pretest, posttest, follow-up at 3 months, and follow-up at 6 months [56]. Participants will be randomly assigned to one of two groups: self-administered program based on positive psychology (Mental Health COVID-19) with assistance via chat or self-administered program based on positive psychology (Mental Health COVID-19) without assistance via chat. Figure 1 shows a detailed description of the study design.

### Study Setting

#### Sampling

Nonprobabilistic, intentional, subject-type sampling will be conducted in the Mexican population according to the following criteria:

#### Eligibility Criteria

The inclusion criteria are age ≥18 years; voluntary participation; access to a technological device (computer, tablet, mobile phone, etc) with an internet connection; a valid email address; basic digital skills in the use of an operational system; and access to an internet browser to answer the initial assessment instruments.

The exclusion criteria are having a psychotic disorder and receiving psychological or pharmacological treatment during the study.

The first removal criterion is not accepting the conditions of the informed consent. Due to the enormous and sudden impact on mental health related to the COVID-19 outbreak, no participants will be excluded from the intervention in terms of the results of their applied psychometric tests. However, the results of the participants who did not fulfill the criteria for any of the measured disorders at the premeasure will be excluded from the statistical analysis. The second removal criterion is absence from the web-based platform for more than 20 days.

#### Sample Size

The sample size was considered based on the effect sizes in controlled clinical studies in which the efficacy of web-based psychological interventions based on positive psychology was evaluated. For the present study, the Cohen *d* index will be used, assuming that the variances of the two groups will be homogeneous; if not, the Hedges *g* index will be used.

Furthermore, the study will include two experimental conditions; a priori analysis was conducted to compare the means between the two independent groups. A conservative approach was adopted, including an effect size with an average magnitude of 0.25 (Cohen *d*, equivalent to *g*=0.5), a significance level (α) of .05 (*P*<.05, which corresponds to 95% confidence) and a conventional statistical power of 80% (1 – β = 0.8). For the analysis, the software G*Power version 3.1.6 [57] was used, and a required sample size of 128 participants was obtained (64 per group).

However, the number of participants will be increased by 30% to control the variable related to dropping out of participants during the treatment; this rate is reported in the literature on web-based treatments [58,59]. Thus, the total required sample size will be 166 participants (83 per group).

### Participant Recruitment

Participants will be recruited through advertisement in digital media (eg, notes in news magazines), as well as through dissemination on social networks. The intervention program will be aimed at adults who can connect via the internet from any part of Mexico. Potential participants can make contact through registration in the Mental Health COVID-19 platform.

### Randomization

Once the evaluation is completed, the users will be randomly assigned to one of the study conditions. The randomization will be performed by an independent researcher using web-based randomization software [60] at a ratio of 1:1 using the method of randomly permuted blocks.

### The Mental Health COVID-19 Web-Based Intervention

The Mental Health COVID-19 web-based intervention aims to provide the target population with a self-administered intervention based primarily on positive psychology; the intervention is aimed at the recognition and development of strengths and virtues through a well-being approach. In addition, it is supported by elements of CBT such as the definition of emotions and the 3-component model of emotional experience. The three components of this model are (1) physiological (what does the person feel in their body that is related to their emotional state); (2) cognitive (what does the person think, where these thoughts are often related to or caused by their emotional state); and 3) behavioral (what does the person do or feel an impulse to do in response to their emotional state) [61,62]. The Mental Health COVID-19 intervention is also supported by the antecedent-response-consequence (ARC) model of emotions [62]. In this model, the antecedent is the event or situation that triggers emotional experiences; these triggers can be something that is happening in the present moment or even something that occurred in the past. Response refers to the responses to emotional experiences, including thoughts, feelings, physical sensations, and behaviors. Finally, consequence can refer to short- or long-term consequences that occurred due to the antecedents and responses [62].

The elements of behavioral activation therapy are also included, such as the importance of physical exercise and the relationship between physical anxiety and its effects on anxiety and depression [63]. The intervention is composed of 15 modules that are adapted to the symptoms that the population may experience during the global pandemic caused by the COVID-19 outbreak. In addition to the positive psychology contents, a module with psychoeducation on grief and loss was added; although this module is not directly related to a positive psychology intervention, the authors considered that these contents will be helpful in providing psychoeducation to patients who suffered the death of a loved one due to COVID-19 or during the outbreak. The combination of components of positive psychology and CBT in a single intervention has demonstrated effectiveness in decreasing the symptomatology of negative affect, depression, and anxiety and in increasing positive affect [64].

A detailed description of each of the modules, as well as the theory and objectives on which each module is based, can be found in Table 1.

**Table 1:** Module objectives of the web-based Mental Health COVID-19 intervention.

Intervention module	Theory	Main objective
1. Understanding our emotions during the COVID-19 outbreak	CBT^a^	Learn about the importance of emotions, including anxiety and why it is experienced [61,62,65]
2. Reflection on preventive measures regarding COVID-19	Positive psychology	Recognize the importance of staying home for the common good [61,66,67]
3. Time for gratitude	Positive psychology	Focus attention on gratitude to reduce the negative impact caused by the outbreak [68-70]
4. To the rhythm of life	Positive psychology	Recognize the importance of a healthy lifestyle [71]
5. Resilience, facing adversity	Positive psychology	Provide tools and recognize personal abilities to recover after a stressful event [72]
6. Helping my mind	Positive psychology	Provide information on the importance of focusing on the present moment with the aim of improving or maintaining emotional balance [73]
7. Taking control	CBT	Define achievable goals to regain a sense of self-control and increase satisfaction during the outbreak as much as possible; decrease avoidance of relevant activities [74]
8. Smile and laugh	Positive psychology	Recognize the importance of laughing and its positive effects on mental and physical health [75]
9. Share concerns	Positive psychology	Recognize the importance of communication with one’s family, friends, and partner and the importance of expressing concerns to loved ones
10. Separated but together	Positive psychology	Recognize the importance of technologies as means of communication to stay connected through telephone calls, chats, and video calls
11. Time to start	Positive psychology	Propose activities that are usually not performed due to lack of time [76]
12. Exercising my mind and body	Behavioral activation therapy	Perform physical exercise involving motor skills of the body and mental exercises that enable the person to stay busy in personal aspects; recognize the importance of sleep hygiene [63]
13. Spirituality	Positive psychology	Provide tools that help develop a level of spirituality to serve as a tool for positive coping with the outbreak of COVID-19 [77]
14. How to deal with grief over the loss of a loved one during the COVID-19 outbreak	Behavioral activation therapy	Provide information about how to cope with the loss caused by COVID-19 or other losses during this time period [78]
15. My inner strength	Positive psychology	Provide support to help the participants focus on their own strengths and know their areas of opportunity [79,80]

^a^CBT: cognitive behavioral therapy.

### Module Delivery Procedure

The contents will be delivered through 15 videos. Each module contains a video with a duration of 10 to 20 minutes, plus homework. The process to generate each of the modules consisted of writing a script based on the theory stated in Table 1 for each module; afterward, the script was narrated by a clinical psychologist with experience in recording video clips and audio capsules. Subsequently, the audio narration was converted to a video clip that included illustrations, short clips, and in some cases, text with explanations of the psychoeducational content. All the videos had the same recording format, in which the narrator provided most of the audio presence and a very small amount of background music was provided without any lyrics. Finally, the videos were uploaded to YouTube, and the privacy option was selected to prevent the videos from being publicly available while they remained accessible with the links integrated in the Mental Health COVID-19 platform. In addition to the videos, the participants could download the exercises indicated in the video as a PDF that could be printed or accessed on the internet; these formats were provided only as review materials for the participants. Video and text elements are among the most common ways to deliver psychological interventions through the internet and can be implemented for a broad range of adult participants; therefore, these two methods were selected. At the end of each video, the participant is asked to answer a 5-question quiz with true-and-false or multiple choice answer options, with the contents observed at the end of each video. It is necessary to complete the quiz with a score of 60% (3 correct questions) to advance to the next module.

The engineering team worked on the usability and accessibility of the platform prior to allowing access to the platform by the general population in terms of responsive design; this ensures that the system has flexibility across both mobile and nonmobile platforms, enabling ease of use across multiple devices [81]. It was confirmed that it is possible to access the intervention through mobile phones, computers, and tablets and properly visualize the contents.

**Figure 2:**
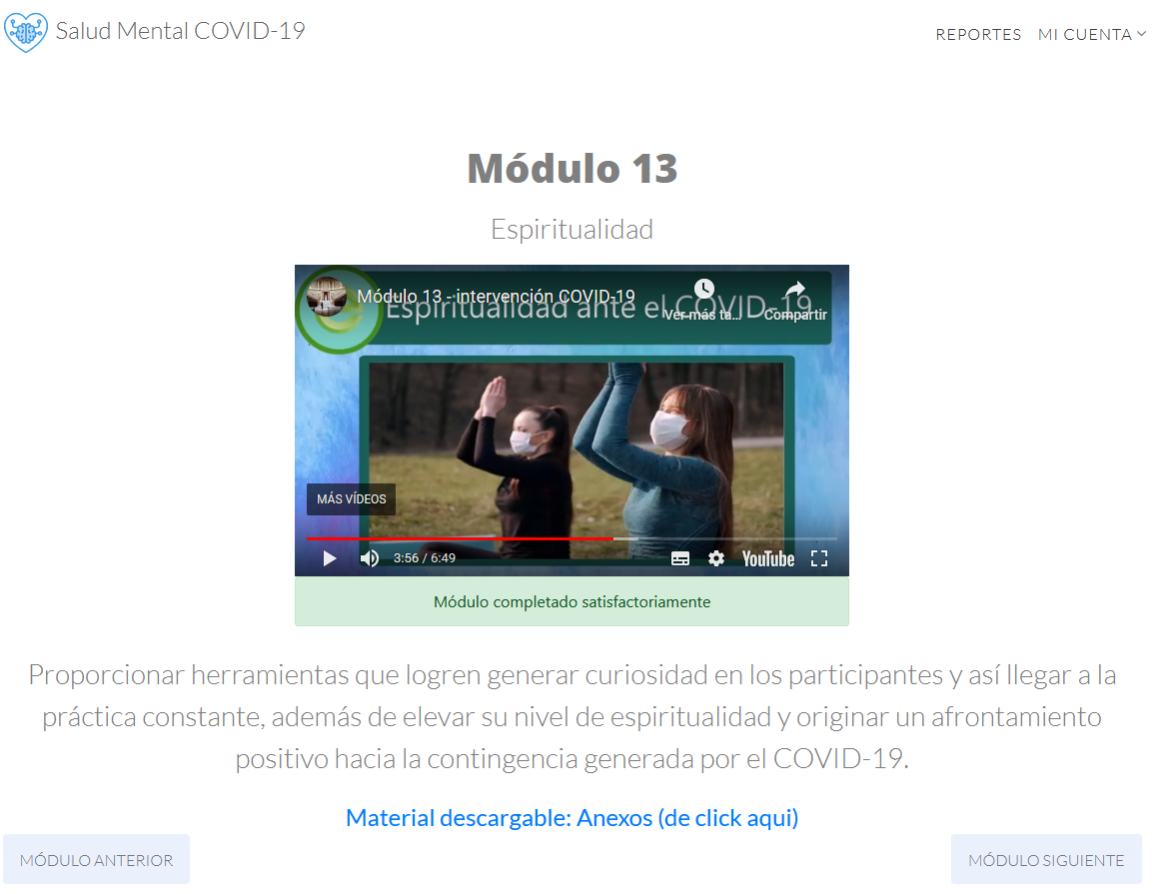
Screenshot of a module of the Mental Health COVID-19 platform (in Spanish).

The modules will be delivered to the participants with a frequency of at least 1 day between modules to give the participants time to integrate the contents and perform the activities assigned to them. Figure 2 shows how the modules will be presented to the participants.

**Figure 3:**
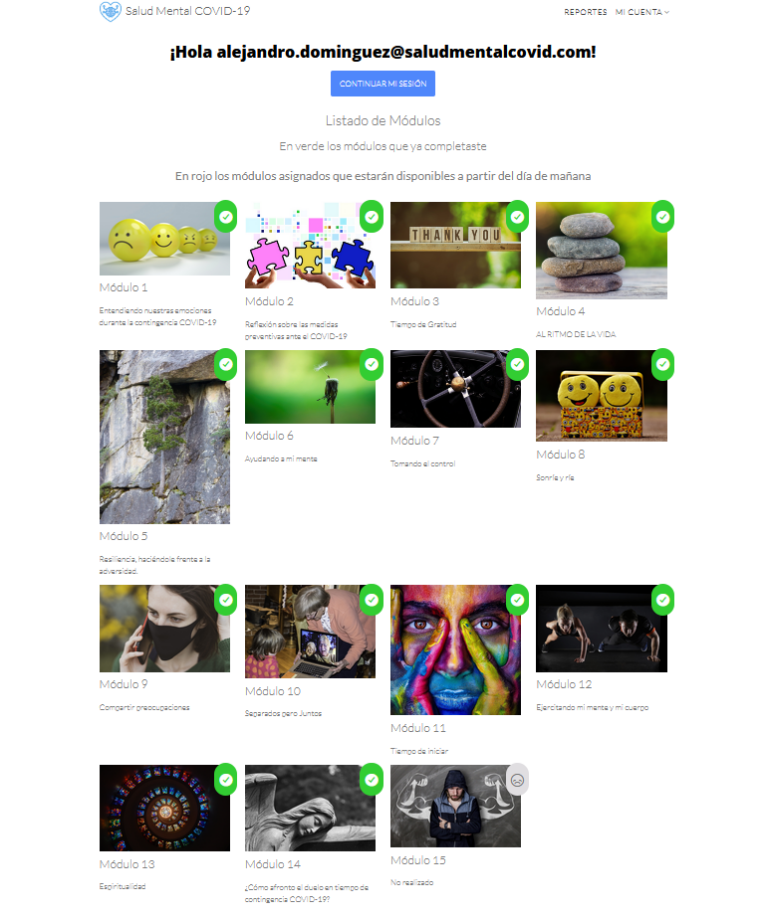
Menu showing the modules completed by the participant.

In addition, the COVID-19 Mental Health platform will include an option enabling the participant to observe their progress and review any modules they have already finished. The modules they have completed will be marked in green. The modules available for completion are marked in gray, and modules that are upcoming or available the next day will be marked in red (Figure 3).

Participants in the Intervention With Chat group may use this tool with unlimited access whenever they log in to the web-based platform, that is, 24 hours per day, 7 days per week. The chat service will be provided through the Tawk app, in which participants will be able to receive help from trained, supervised, and clinically experienced psychologists. The main purpose of the chat is to provide emotional containment in cases of emotional distress; give technical guidance on the platform’s operation; provide psychoeducation in case the participant has doubts about the content of the modules; support the participants in solving problems or encourage them to finish the modules; and provide a referral whenever a participant shows a need for specialized intervention after an assessment, such as substance abuse, depression, self-harm, suicidal behavior, or psychosis. In these cases, the participants will be provided with contact information they can use. Furthermore, a follow-up will be scheduled with anyone using the chat to determine if they have any doubts about the module’s content or about the suggested solutions as well as to explore if they were able to establish contact with the guidance provided.

### Technical Details of the Platform

The main technical objectives of this platform were to separate the parts that make up the system, achieve better code administration, implement the best security techniques, and achieve efficient development; therefore, the web-based system was developed in Visual Basic .NET language under the paradigm of object-oriented programming. The platform is composed of classes and data structures that are assembled in a three-layer architecture. The visualization layer managed through the HTML markup language supports the dynamism offered by jQuery and different support libraries, and communicating with the “Business Rules” layer through asynchronous calls; the platform takes advantage of the facile integration offered by the Active Server Pages (ASP) ASP.NET language and its benefits as a simple syntax language that, in turn, manages all interaction with the data layer, which is managed by a Microsoft SQL server with a relational database.

All development was managed with the Git version manager, and the continuous integration and continuous delivery methodology was used to ensure the quality of the code at the time of its deployment in the production environment and, finally, its availability on the internet.

### Synchronous Writing Conversation Assistance and Monitoring of Psychological Counselors

**Figure 4:**
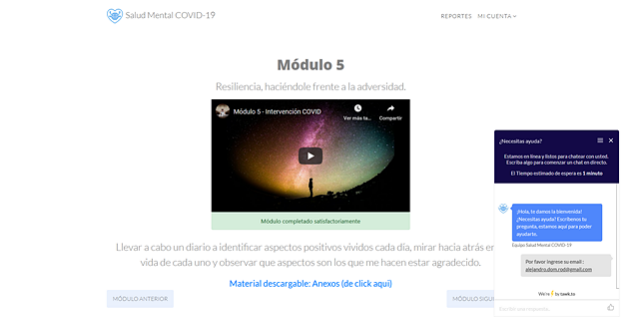
Screenshot showing the integrated chat in the Mental Health COVID-19 platform (in Spanish).

In the case of the intervention condition with synchronous writing conversation assistance, also known as chat, each user will be assigned to a trained psychologist with experience in clinical practice, who will receive prior training on the Mental Health COVID-19 intervention. The function of the psychological advisors is to motivate, guide, and listen to the questions and comments of each participant, providing support with the modules of the applied intervention or brief counseling. Previous studies have shown positive postintervention gains using this resource [82]. An example of the synchronous writing conversation integrated in this platform can be found in Figure 4.

### Measures

All instruments used over the course of the study are self-report questionnaires that are completed on the internet and have psychometric properties regarding the evaluated population. Table 2 gives an overview of all the questionnaires with the time points of the assessments.

**Table 2:** SPIRIT (Standard Protocol Items: Recommendations for International Trials) table displaying the schedule of enrolment, interventions, and assessments in the study.

Time point			Study period							
				Enrollment	Allocation		Postallocation			
				t0	0		t1: Pretest	t2: Posttest	t3: Follow-up 1	t4: Follow-up 2
**Enrolment**										
	Eligibility criteria			✓						
	Informed consent			✓						
	Allocation				✓					
**Interventions**										
	Positive psychology intervention with chat									
	Positive psychology intervention without chat									
**Assessments**										
	**Primary outcome measures**									
		Scale of Posttraumatic Stress Traits		✓				✓	✓	✓
		Widespread Fear Scale		✓				✓	✓	✓
		Urban Strategies Coping Strategies Scale		✓				✓	✓	✓
		State-Trait Anxiety Inventory		✓				✓	✓	✓
		Scale for Suicide Ideation		✓				✓	✓	✓
		GAD-7^a^		✓				✓	✓	✓
		BDI-II^b^		✓				✓	✓	✓
		Pittsburgh Sleep Quality Index		✓				✓	✓	✓
		Positive Psychological Functioning Scale						✓	✓	✓
	**Secondary outcome measures**									
		Opinion questionnaire about the treatment						✓		
		System Usability Scale						✓		

^a^GAD-7: Generalized Anxiety Disorder 7-Item Scale

^b^BDI-II: Beck Depression Inventory second version

#### Primary Outcome Measures

1. Scale of Posttraumatic Stress Traits in Mexican Youth Exposed to Social Violence, validated by Pineda et al [83] and Chávez-Valdez et al [84]. This scale measures posttraumatic stress disorder (PTSD) symptomatology based on version 5 of the Diagnostic and Statistical Manual for Mental Disorders (DSM-5) criteria. This brief scale consists of 24 items that assess traits that support a diagnosis of PTSD. It is answered through a self-report process. It has internal consistency, with a Cronbach α coefficient of .97.
2. Widespread Fear Scale [85], adapted by Chávez-Valdez and Ríos Velasco [86]. This scale measures fear of adversity in a particular context and the feelings it disseminates, as well as other economic and social fears; in this case, it has been adapted for the COVID-19 pandemic. The scale is composed of seven items with options from 0 (nothing) to 3 (a lot), and it has shown an acceptable internal consistency of α=.90.
3. CIU (Cuestionario de Inseguridad Urbana) Urban Strategies Coping Strategies Scale [87], adapted by Chávez-Valdez and Ríos Velasco [86]. This scale is composed of four factors: affective components, physiological activation, cognitive confrontation, and behavioral promotion. In a reliability analysis using the Cronbach alpha coefficient performed by Vuanello [87] in Argentina, a Spanish-speaking country, a Cronbach alpha of .92 was found. A validation of this scale with the Mexican population has been performed by the authors in this manuscript and has been submitted for publication.
4. State-Trait Anxiety Inventory (Spanish version by Spielberger and Díaz-Guerrero) [88]. This instrument measures the symptoms related to anxiety in general (anxiety trait) or how respondents experience anxiety at a certain time (anxiety state). It is composed of 40 items, 20 for state, and 20 for trait.
5. The Scale for Suicide Ideation [89]. This scale aims to assess the frequency of attitudes, behaviors, and plans to commit suicide. It is divided into 19 items with response options of 0-2, giving a total of 0-38, where a score ≥10 indicates an existing risk of suicide. This scale has been validated by González-Macip et al [90] in the Mexican population, obtaining a Cronbach alpha of .84. For the purpose of the analysis, the last item was removed because it evaluates suicide attempts and not ideation; however, with only 19 items, the same Cronbach alpha of .84 was obtained [91].
6. The Generalized Anxiety Disorder 7-Item (GAD-7) scale [92]. This is a brief scale consisting of 7 items designed to measure the severity of symptoms of generalized anxiety disorder. The answers are based on the symptoms perceived during the last week. The questions in this scale are answered in a Likert format with scores from 0-3, where the maximum total score is 21. A score between 0 and 4 points indicates that anxiety is not perceived, and a score between 15 and 21 is an indicator of perceived severe anxiety. The version by García-Campayo et al [93] was used for this study.
7. Beck Depression Inventory second version (BDI-II) [94]. This self-administered scale measures the presence and severity of depression symptoms in adolescents and adults. It contains 21 items with response options on a Likert scale from 0-3, except for items 16 and 18, which have seven response options each. The score ranges from 0-63, where a total of 0-13 indicates minimal depression, 14-19 indicates mild depression, 20-28 indicates moderate depression, and 26-63 indicates severe depression. Studies of the psychometric properties of the Spanish version of the BDI for the Mexican population were conducted by Jurado et al [95] and González et al [96] for version II (Cronbach alpha values between .87 and .92).
8. The Pittsburgh Sleep Quality Index [97]. This instrument evaluates sleep patterns that differentiate people with poor sleep quality from people with good sleep quality. In this scale, seven areas are evaluated: sleep duration, sleep disturbance, sleep latency, daytime dysfunction due to drowsiness, sleep efficiency, overall quality of sleep, and use of sleep medication [97]. The evaluation in the Mexican population showed solid reliability (α=.78) [98].
9. The Positive Psychological Functioning scale. This scale consists of 11 psychological resources: autonomy, resilience, self-esteem, purpose in life, enjoyment, optimism, curiosity, creativity, humor, environmental mastery, and vitality. All of these resources are grouped into a second order factor called Positive Psychological Functioning. This measure has adequate validity and reliability in the Mexican population (α=.91) [99].

#### Secondary Outcome Measures

##### Acceptance, Satisfaction, and Usability Measures

1. Opinion about the treatment [42]. This questionnaire is composed of four questions that report the participants’ level of satisfaction with the treatment and if they would recommend the treatment to a friend or family member, if they consider the treatment useful, and if they think that the treatment was difficult to manage or aversive. The questions are answered on a scale from 1 (nothing) to 10 (very much).
2. System Usability Scale [100]. This instrument is designed to validate the usability of a system; it is composed of 10 items, which are answered on a 5-point Likert-type scale with respect to the degree of conformity of the product (1, completely disagree to 5, completely agree). To obtain the global score of this scale, all the obtained values must be added together and multiplied by 2.5; this will result in a number between 0 and 100, which will be the global value of this scale.

### Data Collection and Management

Due to the structure of how the platform is built, it is possible to know if the participants have not logged in to the platform in more than 3 days. For this purpose, we will consider sending a generic email to all the participants reminding them about the benefits of continuing with the intervention [101].

All the participants may withdraw from the treatment at any time for any reason they consider relevant to interrupt the intervention. The participants will not need to notify any member of the project about their withdrawal from the intervention; however, the main contact points such as email or therapists in the chat will record any notification received about the withdrawal of any participant, and this will be analyzed at the end of the study. Moreover, the structure of this web-based intervention provides data about the users’ behavior on the platform in terms of how often they use it, for how long, and which modules they review, while respecting the confidentiality and anonymity of the users at all times because sensitive data are not requested, nor is it possible to identify the users. These data will allow the researchers to identify if variables such as education level, age, gender, or symptomatology are related to a higher or lower frequency of use of the platform.

### Statistical Analysis

Descriptive analyses will be carried out to characterize the study sample based on demographic variables such as age, sex, occupation, and residence. The abandonment data based on experimental condition, region of the country, and sociodemographic characteristics will be considered. To analyze the clinical indicators, the intensity of symptoms, their duration, and comorbidity with other psychological problems will be reviewed, as well as measures focused on the enhancement of positive emotions, strengths, and virtues.

To determine the differences in sociodemographic and diagnostic variables that could affect the efficacy of the study between the two treatment groups (assisted by psychological counselors via chat or without assistance), statistical analysis will be performed before the intervention.

The Kruskal-Wallis test, with a level of significance of *P*≤.05, will be calculated for categorical variables through chi-square test analysis. The results will be presented in three sections. The first section is contrast analysis to measure the efficacy of the interventions. In this regard, specific measures of anxiety and depression symptoms and positive psychological functioning will be analyzed before and after the COVID-19 Mental Health treatment program. The second section analysis of moderating variables (coping strategies), and the third section measures the acceptance and satisfaction as well as the usability of the system.

### Power

To determine the efficacy of the intervention program, a repeated-measures analysis of variance will be computed using SPSS (IBM Corporation), in which we will compare the pretest measures against the posttest measures in the two experimental conditions. The results will be assessed by performing effect size analyses for each intervention group and between treatment groups (unassisted and therapist-assisted) using the G*Power version 3.1.6 software [57]. A conservative approach will be adopted, and the size effects will be estimated using the Cohen *d* index with a significance level (α) of .05 (*P*<.05, which corresponds to 95% reliability), which will be estimated with a conventional 80% power statistic (1 – β = .8).

### Confidentiality and Ethical Conditions

This study will strictly adhere to the guidelines expressed in the American Psychological Association Code of Ethics for Psychologists [102]. The project supervisors will protect user confidentiality and interaction records during chat support. All participants must read and accept the informed consent, which details the objectives of the study, and then proceed to respond to the evaluation instruments that will provide support to evaluate the effectiveness of the intervention. These instruments are included within the Mental Health COVID-19 platform; therefore, it will not be necessary to provide access to links to other servers, thus protecting the identity and data of each user. At all times, the participants’ rights to confidentiality and privacy of personal data will be respected. The personal data of the participants will be protected for consultation only by the study researchers, and the users may request that their data be removed from the registry and abandon the study at any time. Participation is voluntary, and the intervention will be free of charge for all members of the adult Mexican population who meet the inclusion criteria for the study.

The platform includes a section on privacy policy and privacy rights, which can be found on the website [103]. This section describes the objectives of the platform and provides information regarding the uses of the collected data, use of cookies, links to third parties, and control of personal information. This study received approval from the *Escuela Libre de Psicología, Universidad de Ciencias del Comportamiento* (Ethics Committee of the Free School of Psychology University of Behavioral Sciences) in Chihuahua, Mexico (reference number Folio 2008) on May 1, 2020.

## Results

The clinical trial is ongoing. As of July 2020, enrollment has been completed. We aim to publish the results of the study in December 2020.

## Discussion

### Primary Considerations

This study focuses on addressing the psychological repercussions of the COVID-19 pandemic, and its objective is to test the effectiveness of a self-administered web-based intervention based on the principles of positive psychology for people who are psychologically affected by the pandemic. The intervention is also supported by elements of CBT, and components of behavioral activation therapy were added. Some reviews have shown that web- and computer-based treatments for depression are effective interventions [43-45,51,52].

The objective of this intervention is to enable the participants to internalize and consolidate what they have learned in each module, improve their sleep quality, decrease the anxious depressive symptoms characteristic of the posttraumatic stress generated by the pandemic, and use the learned content as coping strategies and skills on a day-to-day basis. It is important to note that it is possible to apply evidence-based treatments through the internet [41]. These treatments reduce the contact time between the patient and therapist; also, due to confinement during the pandemic, this treatment would not be possible otherwise.

Thus, with the implementation of positive psychology modules, it is expected that the participants’ negative affect and anxiety will decrease significantly and that their positive affect will increase; this would be in accordance with the study by Mira et al [104], in which they suggest that positive psychology techniques can have an impact on clinical symptoms and highlight the need to include these techniques to achieve changes in measures of positive functioning. Also, in interventions based on gratitude, improvements in anxiety, depression, and optimism have been found [29,105].

Particularly, the behavioral activation modules can help users pay attention to the activities they perform daily and realize how this affects their emotional state with regard to their stress level and coping capacity. Quintana et al [106] affirm that strategies to increase physical activity can increase adherence to healthy lifestyles and improve some psychological variables, such as quality of life, quality of sleep, and anxiety. In the same way, the aim of the CBT modules with components of emotion will be to decrease sleep disorders, such as insomnia and nightmares, and decrease the symptoms of PTSD present in the study sample [107] as well as reduce their symptoms of anxiety and depression [108].

The discussion of the study will be in line with the considerations highlighted by Botella et al [109] when evaluating a psychological intervention. When considering the results, attention will be paid to both the axis of efficacy and internal validity. This will enable analysis of the available evidence from the study against alternative explanations and in the axis of effectiveness implied by the generalization or external validity of the intervention, in terms of the feasibility of applying the intervention in various social and cultural contexts of individuals as well as the associated benefits of its dissemination in the context of the health crisis derived from the COVID-19 outbreak.

To summarize, this platform will offer the possibility of reaching a large number of people, reduce costs because it will be administered at home, and offer useful tools for the mental health care of the Mexican population. The web-based methodology offers the possibility of interacting creatively with vignettes, videos, audios, etc; therefore, it is more attractive than traditional interventions.

### Limitations

It should be noted that this study has some weaknesses, such as the lack of ease of internet management for some people, especially older people; also, the dropout rate may be higher than in traditional therapy [41]. In addition, a high dropout rate is observed in web-based interventions performed by the participants themselves, and the influencing factors on treatment adherence and sabotage that may appear during the course of therapy are unknown [41]. It is to be hoped that web-based self-administered treatment will become a generality in the therapeutic community.

## Appendix

Multimedia Appendix 1 Detailed module contents.

## Abbreviations

ARC: antecedent-response-consequence
ASP: Active Server Pages
BDI-II: Beck Depression Inventory second version
CBT: cognitive behavioral therapy
CIU: Cuestionario de Inseguridad Urbana
CONSORT: Consolidated Standards of Reporting Trials
DSM-5: Diagnostic and Statistical Manual for Mental Disorders version 5
GAD-7: Generalized Anxiety Disorder 7-Item Scale
PTSD: posttraumatic stress disorder
