# Supplementary material for: A Self-Administered Multicomponent Web-Based Mental Health Intervention for the Mexican Population During the COVID-19 Pandemic: Protocol for a Randomized Controlled Trial
Source: JMIR Res Protoc. 2020 Nov 16;9(11):e23117. doi: 10.2196/23117 (PMC7704282; doi:10.2196/23117)
Supplement: Multimedia Appendix 1 [file resprot_v9i11e23117_app1.docx]

**Table 1.** Contents and activities of the online intervention “Mental Health COVID-19” modules.

| **Intervention modules** | **Theory** | **Main objective** | **Module content** | **Module activities** |
| --- | --- | --- | --- | --- |
| **1.Understanding our emotions during the COVID-19 contingency** | CBT | Learn about the importance of the emotions, including anxiety and why it is experienced. | Psychoeducation about emotions.  Difference between the Coronavirus anxiety or actual virus symptoms [62]. | The 3 components of emotions [58].  ARC model of emotions [59]. |
| **2. Reflection on preventive measures regarding COVID-19** | PP | Recognize the importance of staying home for the common good. | Brief explanation of what the coronavirus is [63].  Introduction to Mindfulness to decrease the anxiety about being in quarantine. | Letter of thoughts, emotions and behaviors experienced by being at home and having to go to work if mandatory [59].  Brief meditation practice [64]. |
| **3. Time for gratitude.** | PP | To focus the patient’s attention on gratitude to reduce the negative effect caused by the contingency. | Definition [65] psychological and physiological benefits of gratitude [66].  Gratitude as a positive emotion and the influence in decision making [67]. |  |
| **4. To the rhythm of life.** | PP | Importance of leading a healthy lifestyle. | Focus on the rhythm of sleep, food, work, exercise, leisure, rest projects at home, and persistence-commitment. | You have the key. Choose a goal that you have desired for a long time and achieve it, observing the factors that may affect its achievement [68].  Calendar. Delimit the time dedicated to each activity (food, sleep, schoolwork, housework and remaining time in exercise, leisure and personal [68].  List. Activities on a common day before the quarantine. Activities that can be done at home during quarantine [68]. |
| **5. Resilience, facing adversity.** | PP | Provide tools and recognize personal abilities to recover after a stressful event. | Psychoeducation, concepts resilience, crisis, coping (problem-solving strategies, emotional regulation strategies). | Self-affirmation exercise that we could tell ourselves on a daily basis if we are in a crisis process [69]. |
| **6. Helping my mind.** | PP | Provide information on the importance of focusing on the present moment with the aim of improving or maintaining emotional balance. | Psychoeducation on emotional exhaustion and Mindfulness. | Appreciating the moment. Mindfulness exercises focusing attention on a fruit [70].  Registration at the moment.  To pay attention to everyday moments for 15 minutes a day [70]. |
| **7. Taking control.** | CBT | Define achievable goals to regain a sense of self-control and increase satisfaction during the contingency as much as possible. Decrease avoidance of relevant activities. | Psychoeducation about the importance of the objectives and the establishment of these. Motivation. How to go from avoidance to activation. | Reflection on questions regarding the effects on mental health derived from the quarantine. What is valuable to me? What have you lost or stopped doing because of depression or anxiety? [71].  Setting goals during the Positive Psychology intervention [71].  Daily monitoring. Activities carried out and scoring the degree of enjoyment and importance of these [71].  Goal setting in 5 vital areas: Relationships, education/career, recreation/interests, body-mind-spirituality, and daily responsibilities [71].  Selection and hierarchy of activities indicating the level of difficulty to carry them out [71]. |
| **8. Smile and laugh** | PP | Importance of laughing and the positive effects on mental and physical health. | Psychoeducation regarding optimism, happiness, humor, laughter therapy. Benefits of good humor and laughter. | Laughter therapy exercises.  Home laugh. Laugh for 15 seconds.  Tiptoe. Stand up and raise your arms at the same time laughing loudly with the syllable HA following with all the vowels [72]. |
| **9. Share concerns** | CBT & PP | Importance of communication with the family, friends, and/or partner. Importance of expressing concerns with loved ones. | The relevance of sharing concerns. How to share these concerns in a focused way. After sharing the concerns, what resources do we have to solve these concerns? | Write about these concerns by answering 3 questions. 1. Does anyone else feel concerned about the same or something similar? 2. If so, how does this make me feel? 3. Has my way of perceiving the situation that worries me changed by sharing it with someone else? |
| **10. Separated but together** | PP | Importance of technologies as means of communication to be able to be connected through telephone, chats, and video calls. | Remember the importance of the healthy physical distance necessary during the contingency and the benefits of following it. The current means of communication allow us to communicate with great ease through audio, video call or text. Remember the importance of not only talking about COVID-19 but other relevant and/or pleasant topics. Remember to maintain communication with older people during this contingency. | Short-term plans. Schedule a call with friends or family.  Games. Play online with loved ones. |
| **11. Time to start** | PP | Proposal for activities that are usually not carried out due to lack of time. | Explanation of the importance of activities such as artistic activity, writing, reading, cooking, watching a series or movie. | Do an activity that had not been done before due to lack of time [73]. |
| **12. Exercising my mind and body** | CBT | Performing physical exercise that allows the motor skills of the body and mental exercises that allow the person to stay busy in aspects related to him or herself.  Importance of sleep hygiene. | Psychoeducation about the importance of exercise, the relationship between physical activity and its effects on anxiety and depression. Importance of sleep. | Record thoughts before doing the stipulated exercise in 5 videos available online: stretching exercise, cardio and abdomen exercise, exercises for arms, biceps and triceps, exercises for legs and stretching after training [60].  Videos with brain gym exercises. |
| **13. Spirituality** | PP | Provide tools that help to develop a level of spirituality and this serves as a tool for positive coping towards the contingency of COVID-19. | Psychoeducation of spirituality, the importance of spirituality, the relationship between spirituality and health, spirituality and prayer, and spirituality before COVID-19. | Recommendations for actions taken towards spirituality such as focusing on doing good for others. Purify the life, forgive and be forgiven, live with joy, trust and be grateful [74]. |
| **14. How to deal with grief over the loss of a loved one during the COVID-19 contingency?** | BAT | Provide information about how to cope with the loss caused by COVID-19 or other losses during this time period. | Psychoeducation about the emotions experienced by the death of a loved one. Explanation of how to experience a "normal" duel. Phases that go through when experiencing a duel. Difference between unexpected or anticipated losses from other types of deaths. How deaths can be experienced during the COVID-19 contingency. Explanation of when a person may be experiencing a complicated duel. | To record the registry of emotions, thoughts, feelings, and actions experienced since the loss of the loved ones due to COVID-19 [75]. |
| **15. My inner strength.** | PP | Provide support to the participant to focus on their own strengths and know their areas of opportunity. | Psychoeducation about the importance of self-discovery, identifying the strengths and weaknesses, what to do with them and reflection on the current situation before COVID-19. | Pay attention to the metaphor on the computer screen [76].  Make a list with strengths and weaknesses [77]. |
